# Natural Biological Solutions for Chronic Pathological Problems

**DOI:** 10.3390/biom14101248

**Published:** 2024-10-02

**Authors:** Ziqi Jin, Adam C. Midgley

**Affiliations:** Key Laboratory of Bioactive Materials for the Ministry of Education, College of Life Sciences, Nankai University, Tianjin 300071, China; 2120241626@mail.nankai.edu.cn

Naturally sourced biomolecules and their derivatives have had significant historical impacts in terms of their biomedical application. The exploration of peptide/protein-based drugs, polysaccharides, hydrocolloids, natural drugs, and phytomedicines has significantly improved our understanding of how these biomolecules influence the biomedical efficiency of disease-specific therapies. However, it is only in recent years that modern scientific and technological advancements have facilitated and reignited interest in naturally sourced biomolecules as effective drug candidates [1], tools for novel biocompatible drug delivery vehicles [2,3], targeted cell-influencing therapies [4], and immunoregulatory therapies [5,6]. Chronic diseases, such as diabetes, non-healing wounds, fibrosis, and neurodegeneration, place a substantial health burden on the patient, a financial burden on healthcare systems, and have an associated socio-economic impact. Hence, there is an urgent need for innovative strategies in the treatment and management of chronic diseases. Given the emerging evidence that naturally occurring biomolecules possess the potential to impact human health and diseases, it is timely and imperative to further explore and build upon their efficacious applications for addressing chronic diseases.

This Special Issue features five comprehensive review articles from experts in the field, providing the reader with an insightful overview of the promise that natural biomolecules have in addressing the prominent and forthcoming medical and scientific challenges in chronic disease management. The review articles summarize the current literature and research landscape, while discussing emerging and promising experimental and clinical directions. They serve as valuable resources, providing substantial reference material to guide researchers in their development of natural therapeutic interventions that target the underlying mechanisms of chronic disease pathogenesis and recovery.

Chen et al. [7] highlight the potential threat of the misuse or abuse of disease-controlling and growth-promoting antibiotics to human health and the animal husbandry industry. In particular, the impact of antibiotic misuse on the intestinal microbiota (or gut microbiome) is highlighted. A consensus is reached stating that the gut microbiota serves as one of the key elements contributing to numerous diseases, including chronic diseases such as metabolic disorders, type 2 diabetes, liver and intestinal damage, and some cancers. Therefore, the regulation of the gut microbiota and the maintenance of a healthy microbiome can serve to improve the health of the host. The authors provide a detailed discussion on the beneficial mechanisms that several plant-derived bioactive compounds elicit on the gut microbiota, the direct and indirect effects on the host’s own cells, and in the regulation of metabolic disorders (blood glucose tolerance and insulin sensitivity) and liver complications. The highlighted compounds include several that have received increased attention in recent years, namely curcumin, capsaicin, quercetin, resveratrol, allicin, catechin, and lignans. The review provides a comprehensive summary of studies that offer evidence for plant-derived bioactive compounds to serve as gut microbiota-friendly substitutes for antibiotics.

Chronic wounds, such as venous leg ulcers, diabetic foot ulcers, or pressure ulcers are non-healing wounds that significantly impact the patient’s quality of life and exert a considerable financial burden on healthcare systems worldwide [8,9]. Moses et al. [10] provide an extensive review of the past two decades in relation to natural product application in the clinical treatment of chronic non-healing wounds. Following their review of clinical trials that used plant-derived natural therapies for non-healing ulcers, a discussion on recently identified beneficial cellular and molecular mechanisms promoted by select natural products is provided. The authors highlight the plethora of available information on natural products regarding toxicity or adverse effects based on their historical use by indigenous populations, which may help researchers in isolating products for in vitro investigation and assisting their entry into clinical trials.

Fungus-derived molecules include breakthrough drugs such as penicillin antibiotics and cyclosporin immunosuppressants. In the wake of fungus-derived compounds entering clinical trials for the treatment of cancers and drug-resistant depression, there has been a re-emergence of interest in the application of fungal products in chronic disease treatment and management [11,12]. The review by Prescott et al. [13] provides a detailed history of fungal drug discovery and an extensive overview of recent discoveries. After their discussion of the ecological interactions and functions of key fungal molecules, the authors highlight the application of these molecules in human medicine. Additionally, the authors mapped bioactive compounds to a taxonomic tree to provide clues for the discovery of the next “blockbuster” fungus-derived drug from previously unexplored fungi species. The authors highlight that fungal drug discovery remains a challenging field, but the improved resolution gained from further analysis of fungal genomic data may lead to the discovery of novel metabolites and biological mechanisms.

Fibrosis is the end stage of many chronic diseases, wherein myofibroblasts and myofibroblast-like cells produce excessive amounts of extracellular matrix components, pro-inflammatory cytokines, and pro-fibrotic growth factors [14]. While the treatment of fibrosis remains a substantial scientific and medical challenge, accumulating evidence suggests that the blockade of the signaling pathways that drive fibrosis or the promotion of the pathways that antagonize fibrosis can be achieved through the delivery of peptides that mimic the bioactivities of the larger proteins they are derived from. Advancements in analytic technologies, computer-aided molecular docking, and techniques for the biochemical isolation of biomolecules has made the exploration of proteins and their derivative peptides more accessible and affordable. Thus, the application of peptides in the place of proteins (e.g., growth factors) has emerged as an inexpensive strategy to block, activate, or target the key proteins involved in regulating fibrosis in various organs. The review by Zhen et al. [15] summarizes the application and prospects of mammalian-derived small peptides and polypeptides as anti-fibrotics, emphasizing peptides that target key pro-fibrotic and anti-fibrotic pathways in myofibroblasts. The review provides a summary of recent research on both newly developed peptides and updates on promising peptide candidates for mitigating fibrosis. In addition to mammalian peptides, the review discusses the prospects of non-mammalian peptide-based therapeutics before providing an insight into the future perspectives of peptide design.

The increasing size of the aging population has led to an increased incidence of neurodegenerative diseases, which predominantly affect the elderly population. Alzheimer’s disease, Parkinson’s disease, and Huntington’s disease are chronic diseases that seriously impact the patient’s quality of life and exert a substantial emotional burden on the patient’s families [16,17]. As active ingredients in many edible and medicinal health products, polysaccharides have emerged as a new paradigm in the treatment of neurodegenerative diseases. The review by Gan et al. [18] summarizes the current understanding of the polysaccharides that elicit neuroprotective, anti-inflammatory, and antioxidative effects in the context of neurodegeneration. Additionally, the role of polysaccharides in restoring the balance of the gut microbiota to mitigate the pathological development of neurodegenerative diseases is explored. The review summarizes the mechanistic actions of polysaccharides at the cell and signal transduction level before providing an expert insight into the remaining barriers facing the development of polysaccharide products for the treatment of neurodegenerative diseases.

Chronic diseases are inherently difficult to recreate using cell, organoid, and pre-clinical animal models. We envision that with the emergence of multi-omics and computer-aided analyses in the context of chronic disease research, the co-development of accurate cell, organoid, and animal models of chronic diseases may be achievable and provide a platform for the high-throughput testing of natural products and their ease of entry into the research pipeline towards clinical translation. In addition, given that chronic diseases are complex and multifactorial, future treatment strategies may require a combination of multiple interventions, including phytomedicines, fungal drugs, peptides, and polysaccharides used as synergistic co-therapies and/or in combination with other chemical drugs. Indeed, this aspect remains largely unexplored due to the singular focal points of most studies. Additionally, translating findings into clinical practice requires further analyses of the structural complexity and optimization, validation of efficaciousness and safety, and an in-depth understanding of the mechanistic actions of naturally sourced biomolecules.

We hope that readers of this Special Issue of Biomolecules will discover a wealth of insightful information that fosters a deeper appreciation for the potential of naturally derived biomolecules and their regulatory actions in the treatment and management of a plethora of chronic diseases. Moreover, we also hope that the reviews featured in this Special Issue will inspire readers to plan and advance their research endeavors within this exciting and promising field.
